# Foreign Summary

**Published:** 1839-11-09

**Authors:** 


### FOREIGN SUMMARY.

### Velpeau's Clinical Lectures on Ophthalmia. No. IX.

#### INFLAMMATORY AFFECTIONS OF THE CORNEA.

*Treatment of Acute Keratitis, continued.*

*Purgatives.*—Purgatives are much employed in the treatment of keratitis, and are classed among internal irritants; improperly so, in my opinion, as it is by no means certain that they act by irritating the intestines. It is much more probable that they act on the system in general, by the quantity of fluid which the mucous membrane of the intestines secretes under their influence. The purgatives which appear to be most frequently used are castor oil, jalap, scammony, aloes, colocynth, &c.; I am, however, at a loss to decide whether theory or experience has induced practitioners to prefer them. I have myself given all these purgatives a fair trial, either alone or combined, and not only am I unable to say whether one merits the preference over another, but I have even some doubts respecting the value of purgatives in general in this affection.—They appear to be useful as adjuvants, when combined with blood-letting, but given alone their efficacy is certainly but slight. In all cases, however, in which the tongue is foul, the breath bad, the abdomen distended, they are extremely beneficial. Indeed, purgatives ought, I think, to be considered more as medicinal agents, to be employed against the disordered state of the digestive organs, than as general remedies likely to act on the inflammatory diseases of the eye.

Among the various purgatives which are used, there are two which have attracted great attention—the tincture of colchicum and calomel. The tincture of colchicum has been principally employed in England, in the rheumatic and arthritic of ophthalmia; that is, in keratitis and iritis. I have often tried it, in doses varying from fifteen or twenty drops to a drachm and a half or two drachms daily. The tincture acts as an irritating purgative, but its purgative effects are exceedingly variable. Sometimes a patient is purged with twenty drops, sometimes a drachm produces no effect whatsoever. Generally speaking, the tincture does not act in doses under a drachm. I now never give less, often more. I believe that it exerts a beneficial influence over the course of the disease, but I have never seen a marked improvement take place from day to day, as some authors assert they have done; and as we have other purgatives the action of which is preferable, I now seldom employ it.

As regards calomel—the panacea of some ophthalmologists for diseases of the eye—I have given it in every possible manner, in doses of six, eight, or ten grains daily, in doses of twenty-four or thirty-six grains every twenty-four hours, divided into fractional doses of two or three grains every hour, combined with opium or with sulphur. In some cases I have administered it so as to produce salivation in the course of three or four days, in others so as only to produce this effect in twelve or fifteen days. The result of all these essays is, that I cannot allow calomel to be as efficacious a remedy as English practitioners represent it to be; I have seldom seen keratitis rapidly modified by its action, and in some cases, although considerable salivation had taken place, the progress of the disease was not arrested. In the greater number of patients to whom I have given calomel, it seemed to produce no effect whatever on the ophthalmia; with some there was slight amelioration, but that amelioration did not seem to me greater than that which the tincture of colchicum and other purgatives generally produce. The efficacy of this remedy has, in my opinion, been much exaggerated; it is in reality, much less beneficial in keratitis, and, indeed, in ophthalmia in general, than is generally asserted. I, therefore, only employ it when the disease resists the action of other therapeutical agents, or when the constitution of the patient, or the state of weakness in which he is, from previous loss of blood, will not allow us to have recourse to depletion. I am also cautious in administering it, as under whatever form mercury is given, its introduction into the system is often attended with disagreeable consequences. It may produce a mercurial stomatitis, or an affection of the mucous membrane of the intestinal canal, which swells, and becomes of a grayish colour; and this affection is rather difficult to cure.

The remedies which are used as alteratives in the treatment of keratitis, are iodine, sulphur, and tartarized antimony.

Tartarized antimony has been much praised in England by Mr. Lawrence and Dr. Mackenzie; they give it according to the method of Rasori, in doses of eight, ten or twenty grains, in the twenty four hours. Some practitioners combine it with sulphur or opium. To form an opinion of this, or indeed of any other therapeutical agent, there are two kinds of data to consult—the facts published by others, and those which our own experience furnishes. When the facts which are brought forward to prove the efficacy of a remedy are carefully examined, it is, in some degree, possible to foresee what will be the result of any essays undertaken to prove how far the assertions made in its favour are correct. In some instances the facts are so striking, that it is impossible not to allow that the remedy is possessed of some efficacy; in others we cannot but question the influence it has had over the course of the disease, when we find, as in the cases published in favour of tartarized antimony, that it had already existed several days before the remedy in question was employed, and that general and local blood-letting, blisters, and setons, were resorted to at the same time. I have myself often tried tartarized antimony, in doses of from four or six to ten or fifteen grains, but do not believe that it exercises any specific action whatever over the disease.—It either acts as an irritant on the intestinal canal, or on the system in general, by giving rise to a copious secretion from the surface of the mucous membrane. I think it is as efficacious a remedy as the tincture of colchicum, or purgatives in general, but not more so; and as it occasions continued nausea, which distresses the patient, I now scarcely ever employ it.

With regard to the tincture of iodine, and sulphur, I have not often had recourse to them, and then only when all other means had failed. The results I have obtained are of a very unsatisfactory nature. Sometimes the patients were better, sometimes they were worse; but it would be difficult to say whether the change was to be attributed to time or the remedy. I must say I place very little confidence in their action.

In conclusion, the general treatment of this disease only offers one really efficacious therapeutical agent, and that is, general blood-letting, unless we consider blisters applied over the eyelids as forming part of the general medication. All the other remedies are merely to be looked upon as adjuvants.

*Local treatment.*—However successful the general treatment of acute keratitis may be, it seldom entirely subdues the inflammation. In most cases, although the intensity of the symptoms may be mitigated, they do not entirely disappear, and it becomes necessary to have recourse to remedies which act directly on the tissue which is affected. Local treatment is not, however, equally serviceable in every form of keratitis; it is indeed more especially on examining the action of topical applications that you will perceive the utility of the division I adopted when describing the disease. When the deeper layers of the cornea are inflamed, their influence must necessarily be very limited, as they do not remain in contact with the cornea sufficiently long to reach the seat of the affection, either by imbibition, or by endosmosis. Some ophthalmologists have been so much influenced by these considerations as entirely to reject local remedies in the treatment of keratitis. This rejection, however, is not warranted by experience, as even in diffuse interstitial keratitis, the application of local remedies is not unfrequently followed by favourable results. In superficial keratitis they are extremely useful, especially when the conjunctiva is inflamed—and you well know that this is nearly always the case—by dissipating the inflammation of the conjunctiva. They also act on the affection of the cornea; this you will at once understand, if you recollect the nature of the vascular communication which exists between the interior and the exterior of the eye. When ulcerations of the cornea exist, more benefit is to be derived from topical applications than any other class of remedies.

Before we commence the examination of the various topical applications which are used in the treatment of keratitis, I must call your attention to a certain number of therapeutical agents which do not, in reality, deserve the name of local remedies, as they are generally applied round the eye or above the orbits; I mean the mercurial and belladonna ointments, the tincture of digitalis, &c. Mercurial ointment has been praised as an external application by those who thought they derived benefit from mercurials used internally. The cutaneous surface of the eyelids, and the integuments which surround the orbit, are rubbed night and morning with a small quantity of the ointment. Thus applied it is often productive of benefit, but it is not, certainly, as efficacious as most authors have asserted. It is now seldom used alone, being generally associated with some preparation of belladonna, digitalis, or opium. These substances are combined with the mercurial ointment, principally with a view to modify the photophobia and epiphora, which form such prominent symptoms in keratitis. It was supposed that these symptoms originated in retinitis, or in the inflammation of the ciliary circle, and by employing belladonna—a substance which acts principally on the nervous system—it was thought that they might be mitigated. These theoretical ideas are, you well know, erroneous, photophobia and epiphora being generally caused by ulceration of the cornea, conseqently the influence of these medicinal agents cannot be very great. The combination of these substances with mercurial ointment is, nevertheless, advantageous when the pain is intense, as is often the case in deep-seated keratitis. The tincture of digitalis may be employed in the same manner, and with the same results.

The most simple topical remedies are emollients, such as linen steeped in cold or warm water, and poultices. The more important are the various collyria and ointments.

In the first stage of keratitis, if the inflammation is very slight, it may possibly be subdued by bathing the eye continually with cold water; but when that period is passed, a compress steeped in warm water, or a light poultice placed between two folds of linen, is more beneficial. Poultices are useful, as emollients, in deep-seated or interstitial keratitis, unattended with ulceration; but in the superficial form of inflammation, or when there is an ulcer, they appear rather to irritate than to do good. It is probable because this remark has not been made, that many practitioners disapprove of them in every case. Ointments are scarcely applicable to keratitis, unless it be to the superficial form when accompanied by conjunctivitis. Owing to the convex form and smooth surface which the cornea presents, they are swept off by the eyelids before they have had time to act on the disease. They are even injurious when the cornea is ulcerated, as they often remain on the ulcerated surface, and acting as foreign bodies, increase the inflammation. This last remark will also apply to the different dry collyria which are employed, such as calomel, the oxide of bismuth, &c.; they are equally injurious when any ulceration exists.—Dry collyria, may, however, prove useful in superficial keratitis, if the cornea is entire; in deep-seated keratitis, on the contrary, they can be of little or of no use.

The liquid collyria employed in the treatment of keratitis are nearly the same as those which are used in conjunctivitis. They are, as we have already seen, extremely numerous, but not more than three or four are worth preserving. The nitrate of silver collyrium deserves here, as in the treatment of conjunctivitis, the preference over all others; it is an exceedingly useful remedy in superficial keratitis, and is even still more beneficial when the inflammation is accompanied by ulceration of the cornea. You must not, however, expect its effects to be as rapid as in that affection; the vitality of the cornea is not as energetic as that of the conjunctiva, and this physiological condition necessarily exercises more or less influence over the affections of which it may become the seat. I shall not enter into any details respecting the composition and the manner of using this collyrium, as I have already spoken at length on the subject when I gave the treatment of conjunctivitis.

The collyria containing the sulphate of zinc,* and the deuto-chloride† of mercury, belong to the same class. They are both useful remedies, but inferior, I think, to the nitrate of silver collyrium. In some cases of chronic ulceration I have, however, known the solution of the sulphate of zinc succeed, when the nitrate of silver collyrium had failed. These preparations are therefore worth retaining, as one may succeed when the other fails. Narcotics constitute another class of topical agents, among which you will find the tincture of belladonna, the concentrated aqueous solution of opium, Rousseau's and Sydenham's tincture of opium. I have repeatedly applied these preparations pure to the eye in acute keratitis, and have found that in five cases out of six the inflammation was exasperated; I therefore look upon them as topicals of a very irritating nature, and not at all adapted to the acute stage of the disease. Although I disapprove of them when pure in acute keratitis, they may, I think, be serviceable if more or less diluted. Thus, in some cases of ulcerated keratitis accompanied by intense photophobia and neuralgic pain, I have derived considerable benefit from a collyrium containing fifteen or twenty drops of Sydenham's laudanum to four ounces of rose water, to which I sometimes add four or six grains of acetate of lead, or twenty grains of extract of belladonna.

Such is the treatment of acute keratitis. We have yet to study the chronic form of this disease, and its various complications, which we will do in our next lectures; you will then, I I hope, have acquired a full and comprehensive knowledge of the pathology and treatment of the inflammatory affections of the cornea.

*Chronic Keratitis.*

Chronic keratitis, though generally confounded by authors with other diseases, and but little known or studied before the publication of M. Mirault's interesting essay (Archiv. Gén. de. Méd. 1834,) is nevertheless, an extremely common disease. In most instances it is merely the consequence of acute inflammation of the cornea, but is not unfrequently met with as a primitive affection.

The anatomical characters of chronic keratitis not being the same when it commences at the centre of the cornea as when it commences at the circumference of that organ, it will be necessary for us to examine them briefly in both forms of the disease.

The instances in which chronic keratitis first manifests itself in the centre of the cornea are not so rare as is generally supposed. Whenever it is occasioned by external injury, this region is nearly always the first affected; and you must be well aware that it sometimes occurs spontaneously in the same part of the cornea, as we have lately had several cases of this nature in our wards. When the inflammation is diffuse, the following are the characters which the cornea presents:—its usual transparency gradually diminishes, and it assumes a palish tint, as if it were obscured by a slight mist. Its surface when examined with a lens appears to have lost its polish, and to be covered with small, half opaque specks; but no vessels can be traced in its tissue. This my own experience enables me to advance with certitude, although many observers, and among others, M. Mirault, have maintained the contrary opinion. M. Mirault, who, in his thesis, stated that minute vessels might be observed in the cornea in this the first period of chronic keratitis, has, however, since then retracted his assertion. Sometimes these symptoms succeed each other with rapidity; sometimes, on the contrary—and this is more frequently the case—they are slow in manifesting themselves. There is no shedding of tears, and the patient feels little or no pain; but the sight is always more or less disordered—a fact which the nature of the lesion at once explains. The disordered state of the visual function is, indeed, sometimes the only symptom which informs the patient of the existence of the disease. The suffusion of the cornea, slight at first, gradually increases as the affection advances; and, unless its progress be arrested, the cornea becomes lactescent, or of a deep opaline colour; and flakes of coagulable lymph are deposited between the lamellæ of its tissue. This change in the transparency and texture of the cornea is generally less apparent as we approach the sclerotica. The friction of the eyes against the palpebræ causes pain; and photophobia, as also a certain degree of epiphora, soon declare themselves. When the affection has advanced thus far, the circumference of the cornea, and that part of the sclerotica immediately adjoining, becoming vascular, it is then impossible to distinguish this form of chronic keratitis from the form which we have yet to examine.

When chronic keratitis commences, as it generally does, at the circumference of the cornea, the principal characters are to be found in the peculiar vascularity of the çonjunctiva, the sclerotica, and the cornea. The vascular zone of which

* ℞. Sulph, Zinci, gr. iv; Aquæ, oz. i.; Mucil. dr. i. M. ft. collyrium.

† ℞. Hydrar, Deuto-chlor. gr. ¼ ad ½; Aquæ, oz. i. M. ft. collyrium.

I spoke when treating of acute keratitis—and which, as you well know, is formed by the vessels of the conjunctiva, and by those of the sclerotica—exists, but is less regular and less apparent. The conjunctival vessels are superficial, small in number, and irregularly distributed, many of them terminating in the superficial lamellæ of the cornea. Those of the sclerotica, which are given off by the ciliary branches, are deep-seated, numerous, run nearly parallel to one another, and converging as they approach the cornea, are lost in its tissue. Although the cornea thus becomes injected, its transparency is not necessarily impaired; for the small red filaments, which give rise to the vascular appearance it presents, may exist without the slightest effusion of lymph being observed along their course. Mr. Travers says that he has never seen them unaccompanied by slight effusion; but if you observe attentively the patients who enter our wards, you will soon find that the cornea may remain perfectly transparent, though vascular. At a later period, however, coagulable lymph is effused on each side of these vessels; and the opaline streaks, which are thus formed, may, by uniting, entirely obscure the cornea.

You must not, however, suppose that the vascularity is always such as I have just described it. The modifications it presents are extremely numerous, and can with difficulty be accurately described. It is only by attentively studying the disease at the bedside of the patient that you can become familiar with the various forms under which it is observed. Thus the vascularity may occupy a portion of the cornea. When this is the case it assumes a semilunar form, and is either situated at the angles of the eye, where the long ciliary arteries terminate, or at the extremities of the vertical diameter of the cornea, where the short ciliary arteries are distributed. In some cases it is superficial, and is then evidently formed by the anterior ciliary arteries, which anastomose with branches of the muscular and palpebral. Sometimes it is deep-seated and general, and as manifest at the centre as at the circumference.

When the vascularity is circumscribed—and this is principally observed after acute keratitis with ulceration—that part of the cornea which is affected may either remain transparent, or assume a whitish or grayish colour. It may also be covered with small granulations, or there may be a thickening of the conjunctiva. This thickening of the conjunctiva differs from pterygium by its irregular termination, and especially by its adherence to the cornea underneath, which is also more or less vascular.

When the vascularity is superficial, the entire cornea becomes covered with small granulations. Examined with a lens, it appears as if it had been strewn with sand. The vessels which appear on the surface interlace as they arrive at the centre, and form a network, which gives the cornea the appearance of a mucous membrane. It is to this form of the disease, when carried to its greatest height, that authors have given the name of pannus, from the fancied resemblance which there exists between the cornea and a piece of red cloth. It seems as if a soft, thin, red, fungous veil had been applied over the anterior surface of the eye. The cornea, however, still retains some degree of transparency between the principal vascular divisions, so that the patients continue to distinguish large objects, although they are not able to discern with precision their form.

When the vascularity is diffuse, general, deep-seated, and interstitial, the cornea retains, during a longer period, its characteristic properties. The vessels being less interlaced do not so soon modify its transparency, and the granulations of its surface are much longer before they become so developed as to impede the passage of light into the eye. The cornea resembles a mirror, crossed in various directions by small vascular filaments, the volume of which seems rather to increase than to diminish as they recede from the conjunctiva. It is before they disseminate in the tissue of the cornea that the vessels of the conjunctiva and those of the sclerotica anastomose. Between the ramifications of these vessels may be seen streaks, or patches of coagulable lymph, which give the cornea a singular appearance. The pupil always remains clear.

This form of chronic keratitis is nearly always combined with the superficial form. It is impossible to assign a limit to its duration, such is the tenacity with which it resists every plan of treatment.

If in addition to these characters, you bear in mind, that in all cases of chronic keratitis vision is more or less impaired, you will be able at once to recognise the disease, whenever it presents itself to your notice.

*Causes.*—The causes of chronic keratitis being the same as those of acute keratitis, acting either with less intensity, or on less irritable subjects, it would be useless for me again to enumerate them. Among these causes, however, there is one which I cannot pass over, as little attention has been paid to it by surgical writers; I allude to chronic granular blepharitis. I have, in several instances, seen the affection of the cornea gradually developed under the influence of this cause—a fact which the contact of the membrane with the diseased eyelid easily explains. In one case, in which the granular state of the palpebræ had existed for three months, the keratitis corresponded perfectly with the diseased portion of the inner surface of the eyelids, diminishing and increasing, as the granular blepharitis diminished or increased. In another case the relation between cause and effect was still more evident. The patient, when he entered my ward, had granular blepharitis of the temporal half of both eyelids; before long he was atacked with keratitis, but on the temporal half of the cornea only.

*Duration and prognosis.*—It is altogether impossible to assign a limit to the duratiou of this disease. It is one of those affections in which the hopes of the medical attendant, as well as of those of his patient, are repeatedly raised and repeatedly disappointed. The symptoms often appear to give way for a few days, when suddenly, in the course of a night, the disease regains the ground it has lost. Even when the treatment employed has proved successful, and the inflammation appears subdued, a relapse is of exceedingly frequent occurrence. Abandoned to its own course, the duration of chronic keratitis is indefinite; it often remains many months in the same state, and may at last terminate by entirely disorganizing the cornea. Suppuration of the whole eyeball, or even of the cornea, seldom, however, takes place. That form of inflammation which commences by the centre of the cornea is the mildest; yet it often gives rise to specks which impair the sight of the patient more or less, according to the situation which they occupy. In the order of their importance, I may next name partial keratitis, and then the superficial form of the disease. When the circumference of the cornea only is vascularized, the inflammation often gives way, but when the vascularity is general, deep-seated and accompanied by the white streaks I have already described, little or no hope of effecting a cure can be entertained.

*Treatment.*—Chronic keratitis, when it has existed for a considerable length of time, and has invaded the entire cornea, is one of those diseases against which all the therapeutical agents we can employ are powerless, and when it does give way, the cure ought sooner to be attributed to nature than to art. It is in vain that you employ the powders, the ointments, the solutions, the collyria, which are often succsssful in the acute form of the disease—they appear to make no impression. Blisters over the eyelids, which one would suppose likely to prove beneficial, seem to have lost their efficacy. Blood-letting, purgatives, and mercurials, are not more successful agents, and annular cauterization with the nitrate of silver, lauded by some practitioners, has been as inaffective, in my hands, as all other remedies.

Some surgeons, seeing how difficult it is to overcome the inflammation, have proposed the excision of that portion of the conjunctiva which surrounds the cornea, with a view to prevent the afflux of blood to the diseased membrane. If, however, you call to mind what I have said respecting the distribution of the vessels of the eye, you will perceive at once that the operation is not calculated to accomplish the end proposed. By excising the conjunctival vessels which ramify on the superficial lamellæ of the cornea only, you destroy but one of the sources from which that membrane is supplied with blood; the ciliary arteries which furnish the ramifications seen in the tissue of the cornea, remaining intact. As might, therefore, be easily anticipated, the result of the operation is, generally speaking, far from favourable. I have tried it in many instances, but without success. Among other cases I remember that of a man who was in my wards some few years ago. I tried purgatives, mercurials, cauteries on the temples, blisters over the eyelids, and every topical application that can be thought of, yet he left me nearly in the same state as when he entered the hospital. Some time after, the circular incision of the conjunctiva was resorted to in another hospital, and the case was published in the medical journals as successful. A month after this, however, he was again in my wards, exactly in the same state as when he left me.

Fortunately the cases we are called upon to treat are not all of this nature. They may be recent, and then often yield to proper treatment.—If the tissue of the cornea itself is inflamed, general measures are indicated. In such cases I generally proceed in the following manner:—I begin by bleeding from the arm, regulating the quantity of blood abstracted by the intensity of the inflammation, and the constitution of the subject; the following day I give a purgative; the fourth day I cup the patient on both temples, and on the sixth day I recommence the same course of treatment, modifying it as circumstances may direct. Cauteries on the temples, so often employed by Mr. Lawrence, and mercurial frictions, may also be employed. By a treatment thus directed, I have often succeeded, when local measures had proved altogether ineffectual. Collyria are, generally speaking, of little or no use in this form of the disease.

When the inflammation lies principally, or entirely, in the superficial layers of the cornea, the treatment is different, topical remedies being then most likely to prove beneficial. Calomel, bismuth, laudanum, and the various resolutive collyria, may be employed alternately. The solution of the nitrate of silver prepared according to the formula which I have given you, more especially deserves the notice of practitioners. It has often been of great service to me when other remedies have failed to produce any effect. Excision of the conjunctiva is likely to prove successful in this form of the disease, unless, indeed, the inflammation also occupy the deeper seated lamellæ of the cornea; the superficial vascularity of the inflamed membrane being supplied, as you well know, by the vessels of the conjunctiva.

It is generally considered necessary in keratitis, and indeed in nearly all other ocular inflammations, to keep the eyes covered, and to place the patient in a room from which light is more or less excluded. These precepts are, however, founded on erroneous views, and are, I am convinced, more calculated to do harm than good. I do not mean to say that, when the eyes are inflamed, they should be exposed to the open air, or to a strong light without any protection, for this would be falling into the other extreme; but that a middle course may be, and ought to be, adopted. When a portion of the conjunctiva has been excised, the measures which I now condemn must be resorted to with a view to prevent, if possible, the inflammation and suppuration of the wound that has been made. I would here mention, that the loss of substance which follows the circular excision of a portion of the conjunctiva is sometimes attended with disagreeable consequences. In one case, in which the operation had been performed, the formation of the cicatrix drew down the palpebral conjunctiva, giving rise to ectropium of the eyelid. In another case in which the inflammation of the cornea appeared to be kept up principally by the vessels of the conjunctiva, excision of a portion of that membrane was followed by suppuration of the cornea, and the total loss of the eye; it would seem as if the nourishment of the cornea had been supplied by the conjunctival vessels alone.—*Lond. Med. Gaz.*

---

*Proceedings of the Medical Section of the British Association for the Advancement of Science.*

*President.*—Dr. Yelloly.

*Vice-Presidents.*—Dr. Johnstone, Dr. Roget, Dr. Macartney.

*Secretaries.*—Dr. G. O. Rees, Mr. F. Ryland.

*Committee.*—Drs. Blakiston, Booth, G. Bird, Mr. W. S. Cox, Drs. Evans, Foville, B. Fletcher, Hastings, T. Hodkin, Mr. J. Hodgson, Drs. J. Johnstone, Pritchard, Mr. J. Russell, Drs. R. S. Sargent, Vose, Mr. R. Wood, Dr. Wale.

After an introductory address from the President, one of the Secretaries read a paper, communicated by Sir David Dickson, containing, 'Abstracts of a remarkable case of Rupture of the Duodenum, and of some other interesting cases.'

R. H. was admitted into the hospital on March 3d, at three o'clock, P. M., and died before midnight. The symptoms were, severe pain in the region of the cæcum and ascending colon, hurried, restless manner, pale, haggard, and anxious countenance, short, hurried respiration, pulse weak, quick, and irregular. Depletion, followed by aperients, had been resorted to, without relief; leeching, fomentation, and warm purgatives, were resorted to without any relief, and at half-past eleven he sunk. It was ascertained, that he had been fighting and wrestling three days before, when he was thrown with violence on the breach of a gun. Post mortem examination discovered the following lesions:—The stomach, bowels, and abdominal cavity were filled with gas, the descending colon was much contracted; a quantity of ingesta had escaped from four perforations in the duodenum, the mucous and muscular coats of this gut were pellucid and attenuated, as having undergone ramollissement and absorption, in consequence of which, the peritoneal coat seemed to have given way, from distension or mechanical violence; the other viscera seemed generally healthy. It is known that sudden death frequently follows violent feats of tumbling and horsemanship, and, if examination were made, probably similar lesions would be found, or might exist without being detected, from the examination not being sufficiently minute; and thus the cause of death may more frequently occur than is generally supposed.

The next case detailed was one of Ileus, with enormous distension of the cæcum, which occupied the situation of the transverse colon. The usual symptoms of ileus were present—viz., obstinate constipation, stercoraceous vomiting, and singultus. The ileo-cæcal valve was much diseased, being thickened, and as hard as cartilage. The cæcum, which was forced upwards, had a black and phaselated appearance, and was enormously distended. A strong membranous band, the product of previous inflammation, extended across the ileum, and firmly connected it with the meso-colon. Another case was one of *intermittent coma* from diseased brain. This case was remarkable, for the alternations of coma and excitement. The *post mortem* examination showed the arachnoid membrane to be opaque, and to be raised up from the brain by a gelatinous deposit. A considerable bloody effusion was found at the base of the brain, proceeding from the rupture of a true aneurism of the anterior artery of the cerebellum, near its junction with the basilar artery. The coats of the artery exhibited distinct ossific deposit—the cerebellum, on the left side, was wasted and softened in structure, and of the appearance of curdy pus. The aorta was found extensively invaded with ossific degeneration;—scales of bone as large as a sixpence being separable from it, and its elasticity much impaired. Other cases of coma were detailed, in which depositions of a cartilaginous and tubercular nature were found in different parts of the brain. In a case of Phthisis, the *foraman ovale* was found open; cyanosis did not exist; and the patient, a pensioner, had completed his due period of servitude, and had risen to the rank of sergeant, without suffering any inconvenience from the free communication between the right and left sides of the heart.

The next case was one of Phlegmonous Erysipelas, occupying the arm and thoracic muscles of left side, and remarkable for its extreme rapidity. Two cases of severe abdominal disease were also detailed. In the last was observed an extensive deposit of semi-cartilaginous firmness, occupying the subserous cellular tissue; the calibre of the descending colon was so contracted, that, when cut across, it appeared as if encircled by a ring of "a dull white, yet glistening fish-like substance," fibriform, and from half an inch to upwards of an inch in thickness.

Mr. Middlemore read a brief notice of the methods that have been used for the removal of capsular cataract, where the opaque capsule remains after absorption of the lens, for the purpose of introducing to the Section an instrument to facilitate the operation of extraction, without interfering with the transparent structure of the eye.—The instrument consisted of a needle, accompanied by a small forceps, the former capable of being withdrawn, leaving the latter to be fixed on the opaque membrane, and then withdrawn through the sclerotic, through which the needle had been introduced.

In answer to a question from Prof. Macartney, Mr. Middlemore stated, that he had not yet practically proved the efficiency of the instrument.

Mr. Middlemore detailed a case in which the operation for artificial pupil was performed with success, and presented the patient to the Section for examination. About three years ago much injury was done to the face from an explosion of gunpowder. After recovery of the other parts, the eyes were found to be in the following condition:—the right was completely collapsed, the left was staphylomatous, the lens adhering to the staphyloma, but transparent; the lower half of the cornea was opaque, the upper half transparent, but vision destroyed, from the closed iris being opposite to the transparent portion of the cornea. The first effort was to remove the staphyloma, which was done by repeated puncturing of it with a fine needle. When the process of removal was so far completed, as to permit the operation for artificial pupil, the iris was drawn through a small section of the cornea: it bled freely; but on the subsidence of the hæmorrhage and irritation, a sufficient and well-defined opening was found in the iris opposite the transparent portion of the cornea. The external portion of the iris was allowed to remain strangulated by the incision. The patient has already in a great degree recovered his sight, so as even to distinguish large print. He is still under treatment.

Dr. Foville (of Paris) presented a paper, detailing the results of his researches on the Anatomy of the Brain. He commenced by urging the advantages of examining the structure of the brain by manual separation rather than by section, and gave credit to our countryman Willis, as being the first advocate of this method. He showed that the spinal marrow consists of two lateral portions; united by two commissures, between which, on the median line, there exists a double layer of white matter, analogous to the ventricle of the septum lucidum. He pointed out a remarkable difference of structure in the lateral parts of the spinal marrow, between the roots of the nerves, which is rendered most evident by maceration in water, after previous maceration in spirit. He next described the medulla oblongata. Tracing the crura cerebri to the brain, he showed them to consist of two parts,—the one going to the thalamus opticus, the other to the corpus striatum, where they constitute the white matter,—passing through the middle of those bodies, at the upper and outer limits of which they divide into three layers,—the superior passing upwards and inwards, meets its fellows on the median line, and forms the corpus callosum; the second, or middle, is expanded in the hemispheres, which it constitutes, by lining the cineritious matter of the convolutions; the third, or inferior, and by far the smallest layer, passes to the outer side of the thalamus and corpus striatum, meets its fellow inferiorly, and ascending with it, forms the septum lucidum.—In addition to these facts, he stated his more recent discovery, of several nearly circular systems of white fibres connecting the expansions of the superior part of the crus cerebri, which, from their connexion with the olfactory and optic nerves, and also with the posterior part of the spinal marrow, appear to be essentially devoted to sensation. He also stated his fully confirmed observations, that the pathological affections of the thalamus influence the movements of the [arm of the] opposite side of the body, as those of the corpus striatum do those of the lower extremity. He noticed a similar connexion between the lesions of the cornu ammonis and the motions of the tongue. He combated the idea that the frontal, parietal, and occipital protuberances, are dependent on special developement of the corresponding parts of the brain, but are rather to be attributed to the distension of corresponding parts of the ventricles. After the reading of the paper, Dr. Foville demonstrated the leading facts alluded to, on the recent brain.

Prof. Macartney said, that his own researches confirmed those connexions of the fibres of the brain pointed out by Dr. Foville; but a more extensive and minute connexion between the different portions could be traced than those now demonstrated. He also stated, that the roots of the spinal nerves, by a beautiful plexus or net-work prolonged on the spinal column, formed its outer wall, and communicated with those spinal nerves above and below. Dr. Foville was aware of those more minute communications alluded to; but on the present occasion he did not attempt to give a complete exposition of the nervous system, as being unsuitable, from its great extent. Dr. Evans stated, that having Dr. Foville's pathological statements, as derived from his anatomical investigations in the 'Dictionnaire de Médecine,' he had minutely examined the cases reported by Lallemand, Rostan, and Andral, and found them to corroborate those views in almost every instance.

Prof. Macartney read a paper 'On the Means of repressing Hæmorrhage from Arteries.' He stated that the barbarities practised by the older surgeons, such as burning and searing, instead of the ligature, proceeded from the adoption of the false theory, that a certain amount of inflammation was necessary to the healing process, and, although modern surgeons are easier satisfied, the theory has been preserved; the continental surgeons, however, insisting on a greater degree of inflammation than the British. Even the ordinary ligature, he observed, has been known to fail, from the injury and consequent inflammation inflicted by it; to obviate this, he was induced to try the effects of metallic ligatures, from observing that such substances frequently remained in the body without exciting any uneasiness. On applying ligatures of leaden wire to the arteries of dogs, he found, after death, that they remained *in situ*, without surrounding inflammation, or were removed by interstitial absorption, the arteries being impervious. The same results were observed when the experiment was made on the jugular veins of rabbits. An improvement was made by Mr. Weiss on the leaden ligature, by substituting soft metal wire, capable of being knotted. He next alluded to the well-known fact of the closure of arteries in lacerated wounds; this he attributed to the rupture of the elastic coat and the elongation of the outer or cellular, so as to present merely a minute orifice, which was closed by coagulated blood, and not by the retraction of the artery, as Mr. Abernethy had supposed. In the treatment of stumps, he thought it a matter of doubt, what arteries must necessarily be tied, when the powers which nature possesses to repress hæmorrhage be considered, and the cut surfaces be treated as an open wound, with cold applications. He related a case communicated to him lately by Mr. Darley, near Bray, in Ireland, in which, after amputation of the hand of a child, the stump was dressed with lint, kept wet with cold water, *and no ligature was applied or required.* This, the Professor deemed to be the first case on record, in which amputation was performed without the application of a ligature ; he related another case, of severe wound of the thigh, in which the femoral artery was opened, and after some delay, was tied, yet hæmorrhage to a considerable amount recurred ; nevertheless, by keeping the wound exposed, and cold (in the form of ice) applied, the bleeding was repressed, and the closure of the wound proceeded to a favourable termination.

Prof. Gibson, of Philadelphia, stated, that twenty-five years ago, Dr. Physick had introduced ligatures of leaden wire, influenced by the same considerations as Dr. Macartney. This he wished to state, not as detracting from the merit or originality of the learned Professor's views, but as corroborating them. Mr. Hodgson agreed with Dr. Macartney, that inflammation was not necessary to the reparation of injuries, but he did not think that what Dr. Macartney called the modelling process, was the usual means nature adopted for the closing of arteries ; but contraction. He thought metallic ligatures might prove injurious, from causing ulceration and sloughing of arteries in their process through the body. He also objected to soluble ligatures, such as catgut, as liable to premature softening. Silk ligatures he thought injurious from their too long detention on the artery. Hemp, as in common twine, he deemed the best. He agreed with Dr. Macartney that the exposure of an artery promoted its contraction, but thought it might prove a dangerous experiment; experiments on healthy animals might lead to inferences not to be applied without danger, to seemingly analogous cases of invalid patients. Prof. Macartney explained, that he did not advocate the metallic ligature in all cases.—Mr. Wickenden detailed a case strongly supporting Dr. Macartney's opinion, as he thought. It was one in which amputation was performed on a patient, whose arteries were extensively ossified: attempts to fix ligatures repeatedly failed, yet by attending to the elevation of the stump, and the application of cold, serious hæmorrhage was prevented.

Dr. Blakiston read a paper 'On the Sounds produced in respiration, and on the Voice.' He commenced by showing that the respiratory sound, coarse and intense when heard in the trachea, became weaker and softer as it approached the periphery of the chest, at which point the sound, during expiration, had almost totally disappeared. The air, in passing along the trachea and bronchial tubes, would meet with solid obstacles, and therefore be thrown into sonorous vibrations at every alteration of direction. The divergence of sound caused by the great sub-divisions of those tubes, and the constant diminution in their calibre, would necessarily tend to soften and weaken the respiratory sounds from the trachea towards the air vesicles. But the sounds produced by inspiration were carried up to the ear, placed on the chest, by the current of air during that act; while that produced by expiration, was carried quite in a contrary direction; hence the difference in intensity. It was next shown, that bronchial respiration, occasioned by solidification of a portion of the lung, did not take place in the tubes leading solely to that portion, as had been supposed by Andral aud Laennec, but that it took place in tubes leading to healthy expansible vesicles ; and the ear being brought into contact with these tubes, perceived the coarse sound of the air passing and repassing in them. It was contended that no sensible part of the sound of vesicular respiration was produced in or around the vesicles, or by the rubbing of the pleura, otherwise it would be heard in expiration; nor in the mouth or fauces, otherwise stertorous breathing would increase its intensity, which it never does. The voice being an instrument of the membranous reed kind, Dr. Blakiston detailed a number of experiments which he had made with different kinds of pipes on the wind-chest of an organ, which led him to conclude that the quality of tone of wind instruments became uniformly more coarse and buzzing in proportion to the strength of the blast, and the thickness and elasticity of their sides ; in other words, in proportion as the instrument itself entered into strong vibration. Some instances of the manner in which interference and jarring was produced between those solid vibrations of the instrument and those of the air contained in it, were then given. It was shown that both kinds of vibration were concerned in the formation of the voice; hence, when heard over the larnyx, it was found to be coarse and intense; in proportion, however, as these vibrations travelled downwards towards the air vesicles, they were deadened, the aërial waves by the opposing current of expiration, and the solid ones by the increasing mass of the non-homogeneous mass of the lungs; and at their periphery, no resonance of the voice could be detected. When, however, a portion of the lung became solidified, the current of expiration leading from it was stopped, and the spongy lung was transformed into a more homogeneous, and, therefore, a better conducting substance ; hence the voice resounded strongly, and its quality became sometimes so coarse as to produce a tingling sensation in the ear.

*London Athenæum.*

